# Spontaneous uterine rupture and extrauterine pregnancy in a bitch year after unsupervised parturition

**DOI:** 10.1186/s13620-025-00319-x

**Published:** 2025-11-26

**Authors:** Anna Domosławska, Anna Rapacz-Leonard, Andrzej Jurczak

**Affiliations:** Department of Animal Reproduction with Clinic, University of Warmia and Mazury, Oczapowskiego 14, Olsztyn, 10-719 Poland

**Keywords:** Uterine rupture, Dog, Parturition, Extrauterine pregnancy

## Abstract

**Background:**

Uterine rupture in bitches is usually detected during parturition or within hours of delivery because of the deterioration of the clinical condition of the animal. In contrast, this novel report presents a confirmed case of spontaneous uterine rupture without clinical signs, which remained undetected until almost a year after natural delivery.

**Case presentation:**

A one year and nine months old female Shih Tzu bitch gave birth to two puppies at home without veterinary assistance. The parturition took place without any apparent complications, and the female recovered well. After 8 months, the owners decided to perform an ovariohysterectomy, and a mass was found in the abdominal cavity at their local clinic, which was suspected to be a teratoma. Approximately 3 months after this surgery, the dog was presented to the authors' clinic for consultation where an ultrasonography and exploratory laparotomy were performed. The mass was found to contain three foetuses (two mummified) and signs of haemorrhage were still visible under the peritoneum. During the surgical procedure, scar tissue was found to extend throughout the uterine wall. These findings led to the diagnosis of spontaneous uterine rupture and ectopic pregnancy. Following removal of the mass and uterus, the bitch recovered well.

**Conclusion:**

Carrying extrauterine foetuses is always associated with risks (peritonitis, septicaemia, development of teratoma, organ damage and subsequent internal haemorrhage). This case demonstrates that even if a bitch gives birth at home without any apparent complications, an ultrasound examination of the reproductive tract should be carried out after parturition.

## Background

The incidence of uterine rupture in bitches is not well known [1, 2] but it is not considered to be common. Darvelid and Linde Forsberg (1994) found no uterine rupture in 182 bitches they examined. Similarly, Stolla et al. [3] did not find this condition in 337 cases of dystocia in bitches they examined. When uterine rupture does occur in this species, it may be associated with mechanical injuries such as road traffic accidents, uterine torsion, fetal death, improper parturition techniques including excessive use of oxytocin, and systemic or uterine infection [4–8]. In other species, like bovine and equine, uterine rupture is mainly a consequence of dystocia and most cases occur following forced traction, uterine torsion, twin pregnancy, fetotomy, or delivery of emphysematous fetuses [9–17].

Uterine rupture in the bitch is usually detected during parturition, or within hours of delivery, due to the deteriorating clinical condition of the animal. It can be diagnosed by taking a detailed history, observing clinical signs and performing additional investigations such as abdominal ultrasonography, radiography, blood tests and exploratory laparotomy [18, 19].To the best of the authors’ knowledge, this is the first report of a confirmed uterine rupture that was not diagnosed until almost a year after a natural delivery.

## Case presentation

A one year and 9 months old female Shih Tzu bitch (6.5 kg body weight), came for consultation at the Clinic of Animal Reproduction at the University of Warmia and Mazury in Olsztyn, Poland, in October 2023. In November 2022, the bitch (10 months old) gave birth to two puppies. This pregnancy was not diagnosed or monitored by a veterinarian and there was no knowledge of the number of puppies to be expected. The bitch did not become pregnant subsequently and as there were no plans for further litters, it was decided that the bitch would undergo an ovariohysterectomy at the local clinic in July 2023. During surgery, a dense mass of brown tissue resembling a neoplastic growth was found between the intestines and the uterus. The intestinal mesentery was covered with a brown coating and hairs. On the basis of external examination alone, the local doctors suspected a teratoma. The decision was reached not to proceed with surgery and the abdominal cavity was closed without removing the ovaries or uterus. The owners were informed that the prognosis for recovery was poor and that they should consider euthanasia if the animal’s health deteriorated. However, the bitch recovered quickly and showed no signs of distress. Therefore the decision was taken to have consultation with another veterinary clinic.

During the general examination, the bitch was noted to be clinically healthy with all vital parameters within normal limits. The owners confirmed that there had been no pregnancies after the birth of the two puppies in November 2022. The mammary glands were not swollen and there was no secretion; the vulva was pale and not swollen. As the female dog was fasted, blood was collected for examination. The results showed no deviation from physiological ranges (basic haematological and biochemical profiles (Table 1)).

**Table 1:** Blood basic haematology and biochemistry profiles before the surgery. Note: only essential haematology parameters are listed due to the extensive number of measured parameters

Parameter	Patient result	Reference range
Leukocytes	6.5	4.9–17.6 G/l
Erythrocytes	8.5	5.4–8.7 T/l
Haemoglobin concentration	20.6	13.4–20.7 g/dl
Thrombocytes	252	143–448 G/l
ALT (GPT)	37	25–122 U/l
Alkaline phosphatase	40	14–147 U/l
AST (GOT)	22	14–59 U/l
Total protein	73	54–76 g/l
Albumin	34	28–43 g/l
Creatinine	78	44–133 µmol/l
Urea	5.2	3.2–10.3 mmol/l
Sodium	150	142–153 mmol/l
Chloride	113	106–120 mmol/l
Potassium	4.7	3.9–5.8 mmol/l

Abdominal ultrasonography was performed with a standard 8 MHz microconvex probe, both with the animal standing, and in dorsal recumbency to allow 3-dimensional pathological assessment. A solitary mass lesion was observed in the cranioventral part of the abdomen (2.4 cm in length, 0.9 cm in width). No fetal heartbeat or vascular flow was detected by colour flow Doppler ultrasound and no amniotic fluid was detected. The detection of skulls and long bones within the mass, confirmed that it contained foetuses. Hyperechogenic fibrous bridges were observed around the masses. It was suspected that the adhesions involved the small intestines, but this could only be confirmed by surgery, which was recommended.

### Surgery

Prior to surgery, the owners consented to an ovariohysterectomy, to avoid any further possibility of uterine lesions. Additional factors which led to the decision to performe ovariohysterectomy rather than ovariectomy included the similar morbidity rates for both procedures, and the reduction in risk for long-term complications such as pyometra or uterine stump abscesses and urinary incontinence [20, 21].

An exploratory laparotomy was performed. Prior to surgery, the bitch was premedicated with dexmedetomidine (Dexdomitor, Zoetis) [0.005 mg/kg i.m.], fentanyl (Fentadon, Dechra) [5 mcg/kg i.v.] and maropitant (Prevomax, Dechra) [0.1 mg/kg s.c.]. Anaesthesia was induced with alfaxalone (Alfaxan, Orion Pharma) [2 mg/kg i.v.]. The dog was intubated, and anaesthesia was maintained with isoflurane in oxygen, adjusting the concentration as needed to maintain a suitable plane of anaesthesia. Due to a high risk of perioperative pain, due to the adhesions found during the ultrasound examination, fentanyl was administered at a constant rate infusion at a rate of 12 mcg/kg/h throughout the entire surgery. Additionally, a constant rate infusion of Ringer’s solution was given at 4 mL per kg per hour until the patient had recovered from the surgery. Intraoperative monitoring included measurement of heart rate, respiratory rate, and rectal temperature. Electrocardiography, non-invasive blood pressure measurement, capnography, and pulse oximetry were also performed.

**Fig. 1:**
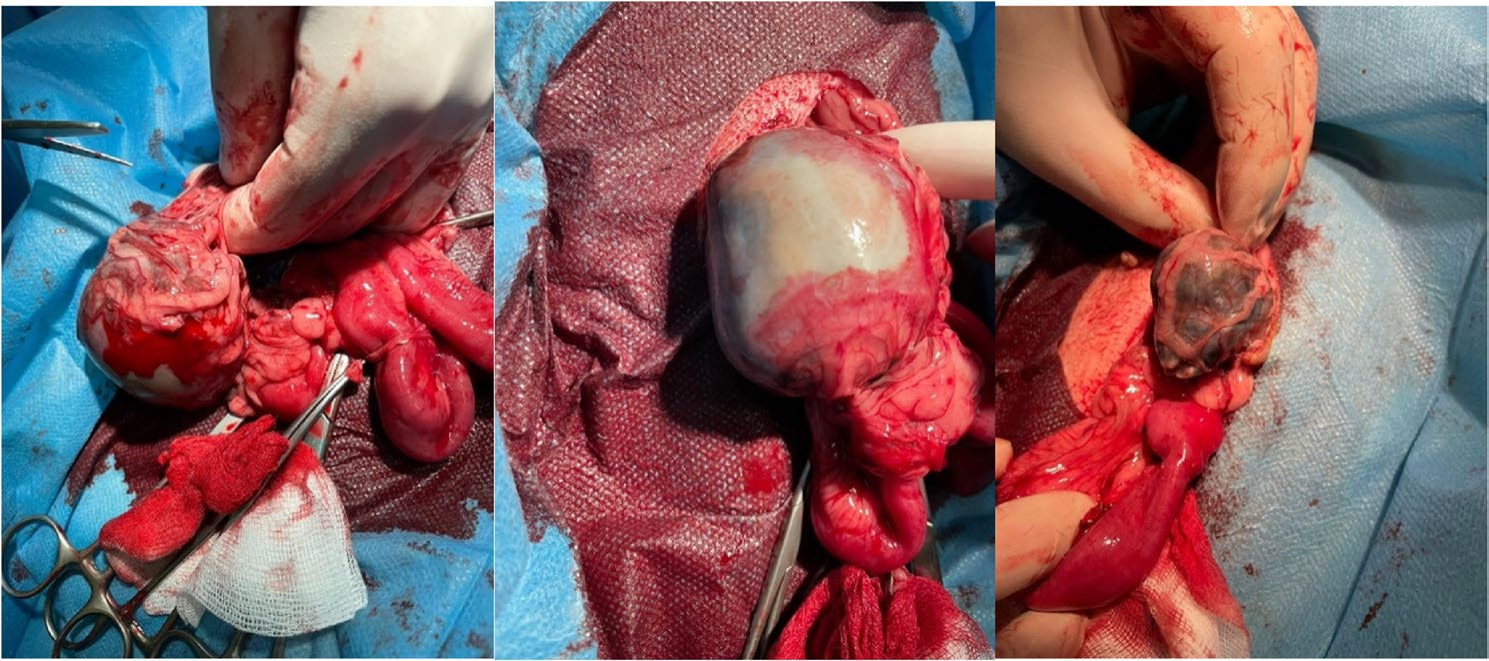
Extracting the foetuses from the abdominal cavity

The ventral abdomen was surgically prepared for midline access. A small midline incision was made caudal to the umbilicus and the abdominal cavity was exposed. Three solitary, dark brown, 6–8 cm long masses were found in the abdominal cavity. All three structures were firm on palpation and were encapsulated and partially adhered to the intestinal mesentery and peritoneal omentum. The adhesions were cut with electrocautery to avoid excessive bleeding. The abdominal cavity was lavaged with Ringer’s solution to remove any remaining hair. Finally, the abdominal wall was closed with three layers of continuous sutures (absorbable PGA 2 − 0, Atramat, Mexico) (discharged home the same day after surgery). After surgery, the bitch received subcutaneous meloxicam (Metacam, Boehringer Ingelheim) [0.2 mg/kg], followed by meloxicam oral suspension (Meloxoral, Dechra) [0.1 mg/kg per os] at 24 and 48 h. Starting on the day of surgery, the bitch received antibiotic therapy (Synulox RTU, Zoetis [7.0 mg amoxicillin, 1.75 mg clavulanic acid/kg i.m.] which was continued for the next five days; Synulox Palatable Tablets, Zoetis [12.5 mg/kg, per os twice daily]. The bitch recovered uneventfully post-surgery and was discharged home the same day (Fig. 1).

### Post-surgery examination

**Fig. 2:**
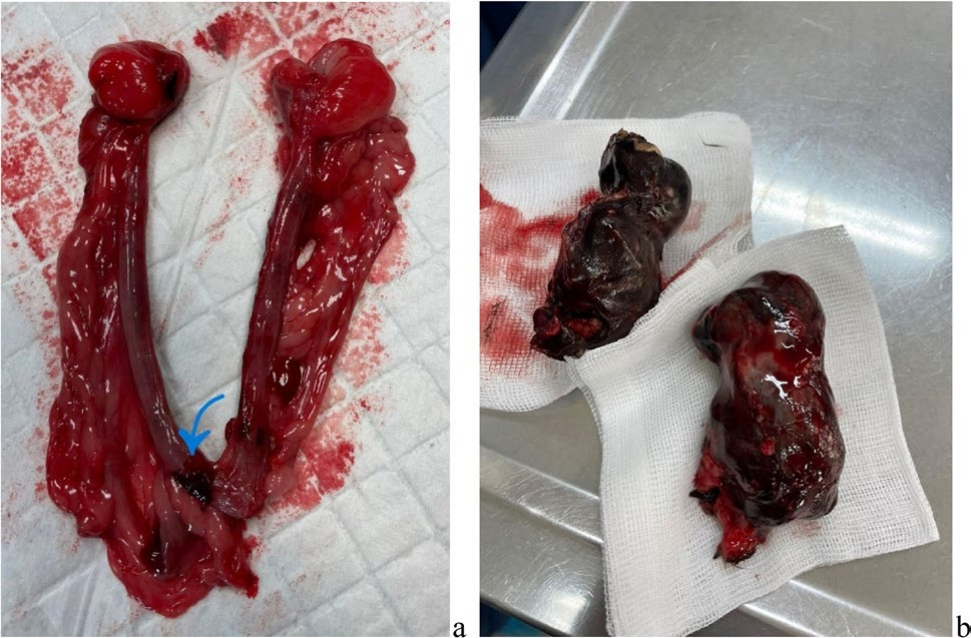
**a** The uterus, following ovariohysterectomy. The blue arrow indicates the site of the suspected rupture at the base of the right uterine horn. **b** Two of the three foetuses, following extraction from the abdominal mass

**Fig. 3:**
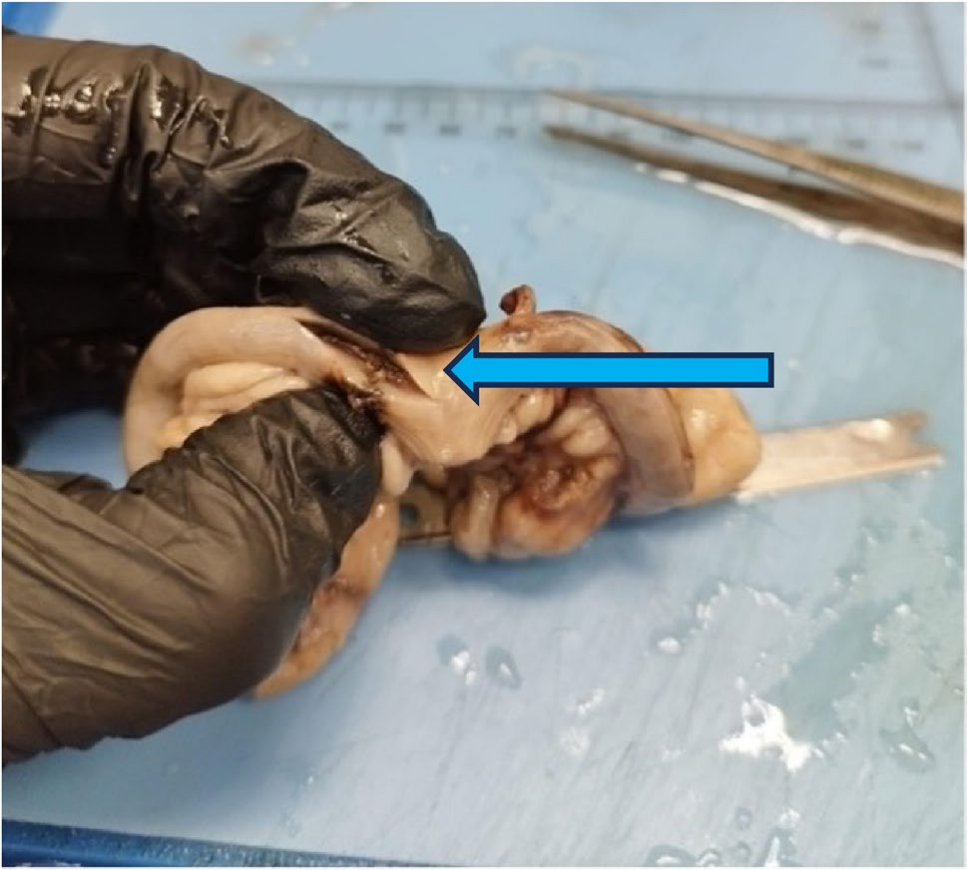
An incision was made in the uterine wall to demonstrate the full-thickness nature of the lesion, which was suspected to be the site of the uterine rupture (blue arrow)

**Fig. 4:**
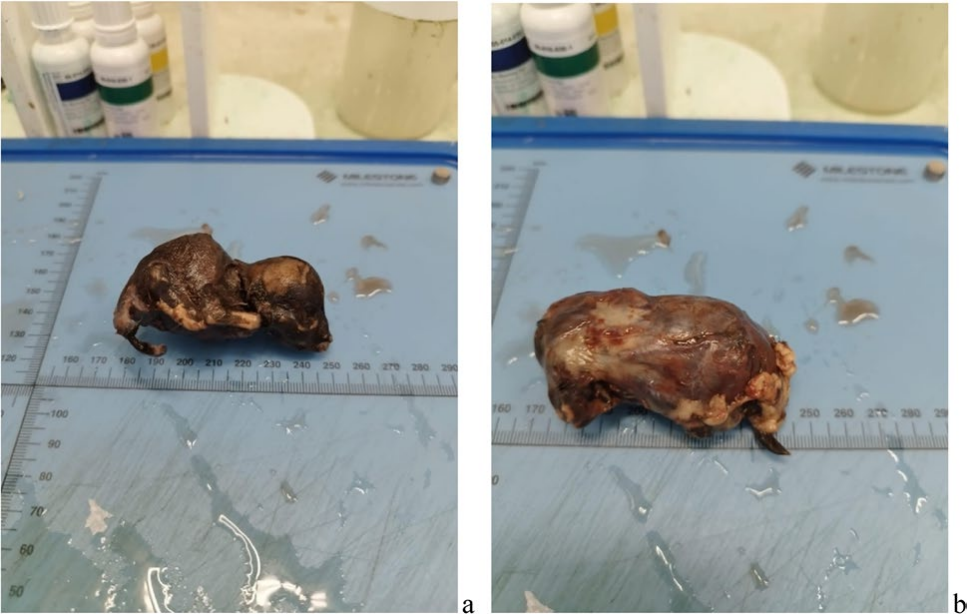
Two of the three foetuses, after removal from the abdominal cavity. **a** shows one of the two mummified foetuses. **b** shows the 3rd foetus, still covered by an intact amniotic sac.

Removal of the uterus revealed that the perforation had occurred at the base of the right horn. The scar penetrated the entire wall of the uterus and signs of haemorrhage were still visible under the peritoneum. No other lesions of the uterus nor any remaining placental or foetal tissue were detected. Two of the three foetuses were mummified; the third foetus still possessed an intact amniotic sac and was therefore better preserved. All of the foetuses showed the level of development that would be expected at the time of delivery (Figs. 2, 3, and 4)

### Histological findings

**Fig. 5:**
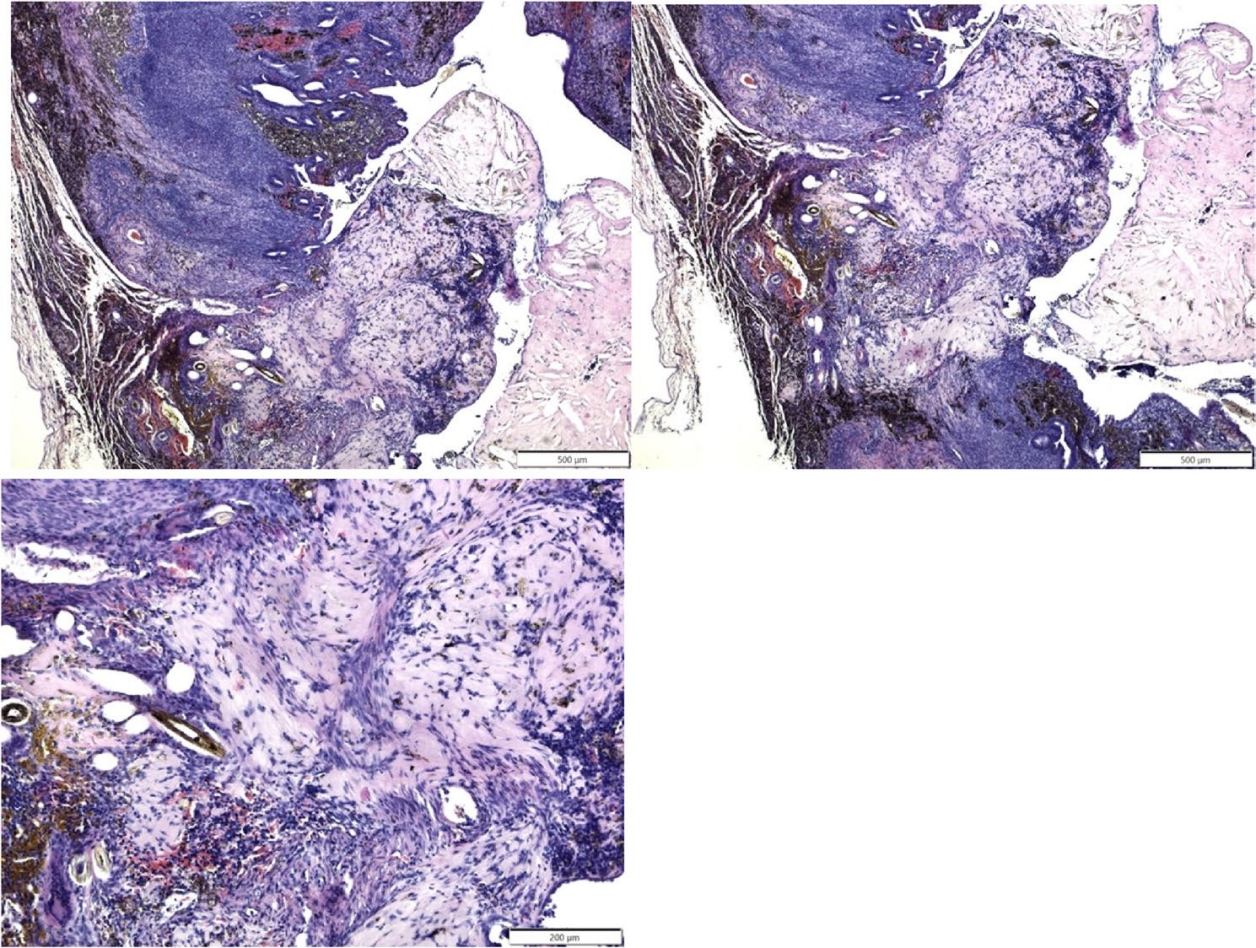
Histological images of scar tissue at the site of the uterine perforation. The connective tissue penetrates the entire wall, indicating that the uterine wall has ruptured completely

Samples were taken from the site of the suspected uterine rupture and were submitted for histology. The histological findings confirmed that the rupture of the uterine wall was complete, extending from the endometrium to the peritoneum. The area of rupture was filled with connective tissue, mainly consisting of cells and collagen, i.e. scar tissue (Fig. 5). In addition, hairs were found embedded in the scar tissue.

#### Discussion and conclusions

To the authors’ knowledge, this is the first reported case in which, following the natural birth of healthy puppies, spontaneous uterine rupture occurred, leading to the death and intra-abdominal retention of the remaining puppies for almost one year. Rupture of the uterus at the time of parturition in bitches is thought to be rare and has only been described by a small number of authors [4–8, 22, 23]. This condition is also uncommon in humans. Ophir et al. (2003) reported that uterine rupture occurred in 0.035% of 117,685 births to Israeli women. The main causes of this condition in these women were previous caesarean section, malpresentation and dystocia in the second stage of labour [24–26].

One reported case of uterine rupture in a bitch occurred in a singleton pregnancy during the spontaneous delivery of a dead, oversized foetus with cranial deformity [4]. The puppy developed in a ventral position; its death and size prevented it from turning to a dorsal position. The bitch was unable to deliver the puppy unassisted and did not receive veterinary assistance in time. The uterine rupture was therefore due to prolonged uterine contractions. In this case report, the authors cannot be sure of the time of death of the foetuses. However, if it was pre-partum, it could have led to thinning and then rupture of the uterine wall, followed by expulsion of the three foetuses into the peritoneal cavity.

In cases of obstructive dystocia, such as those reported by Hajurka et al. (2005) and described by Johnson et al. (2001a), obstetric interventions must be undertaken with caution and caesarean section is the preferred method of resolution [27]. Excessive doses of oxytocin can cause uterine canal obstruction, uterine spasm, discomfort and, in extreme cases, uterine rupture, and foetal injury or death [1, 27].

Humm et al. (2010) described a uterine rupture in a Great Dane bitch. The dog failed to whelp naturally, so oxytocin was administered in conjunction with manual assistance. The uterine rupture may have occurred after this obstetric assistance. It should be noted that there was no oxytocin overdose in this case and all puppies were delivered without caesarean section. Manual assistance is not possible in most bitches because the animals are too small, but it can sometimes be performed in large breeds such as Great Danes [6]. It has been suggested that the use of forceps may cause uterine rupture, but this is based on anecdotal evidence and no such cases have been reported in the veterinary literature.

Another case of uterine rupture was described in an Indian Mongrel bitch and was associated with infection and trauma inflicted during assisted manual delivery during sequential whelpings [7]. Uterine rupture was attributed to foetal maceration as a result of systemic disease in the pregnant animal, and a population of mixed bacteria was found in the fluid collected by abdominocentesis. Rosenberg et al. [28] reported uterine perforation stated 4 days postpartum associated with prolonged labour, retained placenta and metritis.

Uterine rupture can also be caused by repeated dystocia in subsequent pregnancies, which can lead to weakened areas of the uterine wall and an incomplete rupture [7, 8, 27, 29].

One previous case was similar to the case described here- a uterine rupture that could have occurred during pregnancy or the perinatal period [22]. In this case, an exploratory laparotomy (performed because of persistent vomiting and suspicion of a foreign body) revealed an encapsulated foetus adhered to the mesentery of a neutered 6-year-old poodle bitch. There was no evidence for peritonitis, or for remnants of uterine or ovarian tissues within the bitches abdominal cavity.

In this case report, the cause of the uterine rupture is unclear. It can be assumed that the puppies died during parturition, as they showed the degree of development expected at term, and most likely underwent mummification after being expelled into the abdominal cavity.

The novel aspect of this case is that the uterine rupture and extrauterine pregnancy were not diagnosed until almost a year after parturition, and the bitch appeared to be in good health during this period. However, there may be other unrecognised cases similar to this one [23]. Carrying extrauterine foetuses always carries the risk of peritonitis, septicaemia, development of teratomas and organ damage due to pressure from the foetuses, which may lead to internal haemorrhage [9, 10, 23, 30–33].

In conclusion, this case suggests that even in situations where a bitch gives birth at home without apparent complications, an ultrasound examination of the reproductive tract should be performed after parturition because of the possibility of undetected uterine rupture and extrauterine pregnancy. Such an examination should be recommended by the attending veterinarian.

## Data Availability

All the data are presented within the paper.
